# Nitrogen management in the optimization of microbial plastic waste upcycling

**DOI:** 10.1186/s40643-026-01046-z

**Published:** 2026-04-11

**Authors:** Kimia Noroozi, Hong Chen, Vanessa M. Hupp, Mark A. Blenner, Robert C. Brown, Zhiyou Wen, Laura R. Jarboe

**Affiliations:** 1 Department of Chemical and Biological Engineering, Iowa State University, Ames, IA 50011 USA; 2 Department of Food Science and Human Nutrition, Iowa State University, Ames, IA 50011 USA; 3 Department of Chemical and Biomolecular Engineering, University of Delaware, Newark, DE 19716 USA; 4 Bioeconomy Institute, Iowa State University, Ames, IA 50011 USA

**Keywords:** Nitrogen, Media optimization, Hydrocarbon utilization, Nonconventional yeast

## Abstract

Bioprocesses grant us with broad opportunities to build a circular carbon economy. Using microbes allows us to replace non-renewable resources and change waste management strategies, including recycling and upcycling. However, research still lags on strategies to enable economic viability of these types of bioprocesses relative to the traditional production methods. Tuning the composition of the microbial growth medium is one way to address these issues. This allows us to gain insight into the interactions of nutrients and the focal organisms in order to formulate appropriate media recipes to support the metabolic needs. This step, which is often overlooked in proof-of-concept research, can substantially improve process metrics. After carbon, nitrogen is the most important and costly nutrient and should be given thorough consideration. In this study, we utilized nitrogen sourcing to tackle the biological upcycling of thermally oxo-degraded plastic waste using a previously described non-conventional yeast with the ability to utilize hydrophobic substrates, *Candida maltosa.* We compare the use of algae extract and casamino acids as organic, amino acid-based nitrogen sources to inorganic ammonium sulfate with the goal of increasing growth metric and biomass production. Our findings show that the use of algae extract and casamino acids promotes 2X–25X higher growth in model compounds and up to 2X on TOD products. Significant changes were observed in elemental composition of the cells in response to the change in nitrogen and carbon source. These changes align with the ~ 3X increase in internal protein content in the case of cells grown on casamino acids and TOD. Membrane properties and fatty acid content of the cells are also largely impacted, with a significant decrease in saturated and unsaturated fatty acid content, resulting in a reduced average lipid length in the case of casamino acid and TOD grown cells. Three proteins, namely the nonspecific lipid transfer protein POX18, the glycine zipper 2 transmembrane domain-containing protein, and the histidine triad nucleotide binding protein HNT1 were expressed in cells grown on TOD. The results from this study highlight the importance of nutrient management in non-model organisms and the biological challenges associated with the utilization of these compounds which can be insightful in future genetic engineering efforts for improved performance.

**Graphic abstract:**

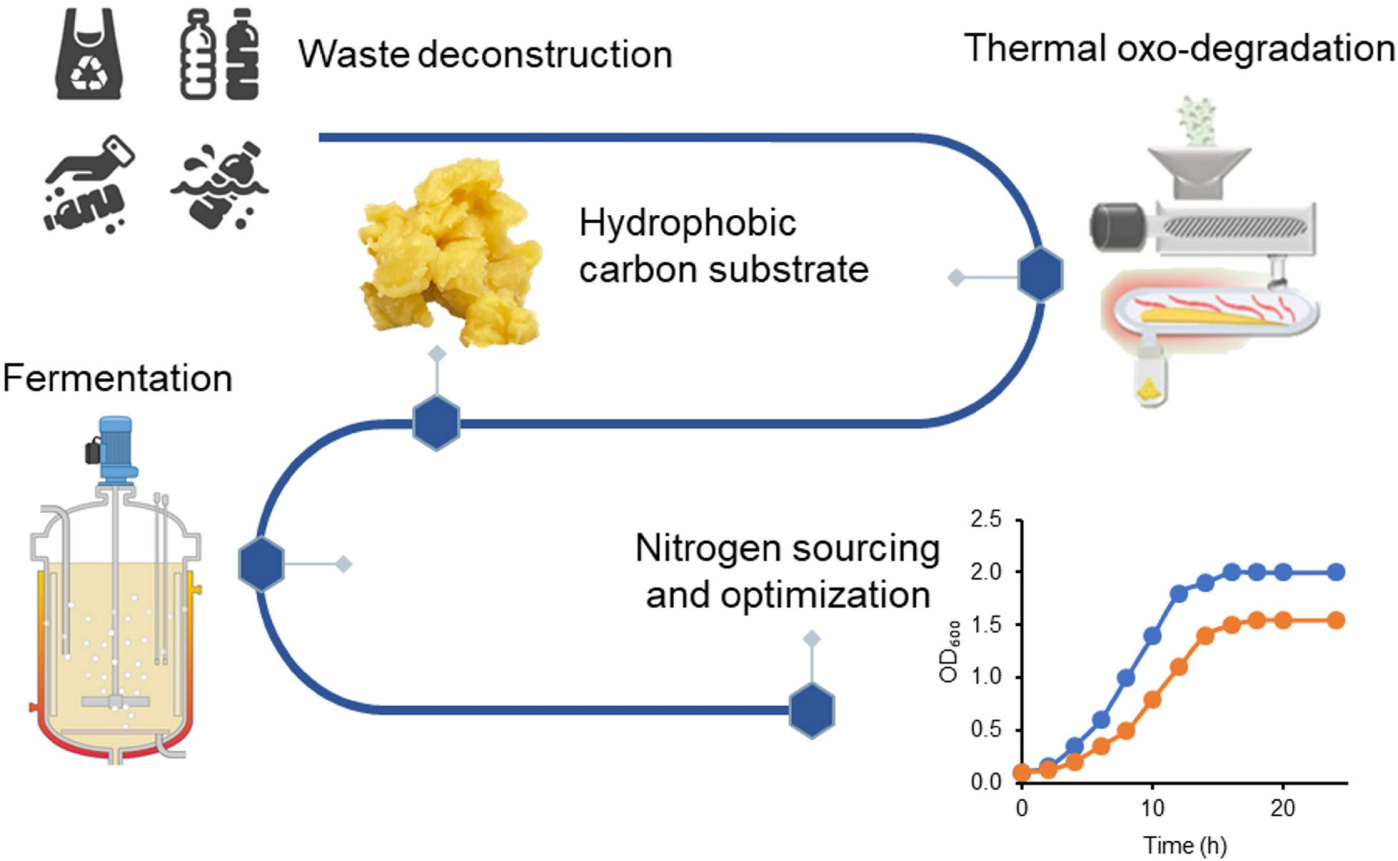

**Supplementary Information:**

The online version contains supplementary material available at 10.1186/s40643-026-01046-z.

## Introduction

As the human population grows, the prospects of climate change, pollution, waste production, and resource depletion have been becoming increasingly dire (Hoornweg et al. 2013). In efforts to offset the negative impacts of human activity, the use of biomass has continued to establish itself as a solution (Leong et al. 2021). This approach, referred to as biorefining, has the potential to strategize the recycling of finite resources and facilitate a circular economy built on renewable materials. Among the various waste streams that can be fed into this biorefinery model, plastics are a major challenge. Their mechanical properties and low cost make them an ideal material for a wide range of applications (Naderi Kalali et al. 2023). However, the same characteristics paired with a lack of waste management strategies have now turned plastics into a serious environmental issue (Naderi Kalali et al. 2023; Pereyra‐Camacho and Pardo 2024). Our increasing depth of knowledge of microbial species and consortia, along with their tools and abilities for plastic waste degradation, set the stage of the possible use of microbial cell factories for addressing the problem of plastic waste (Gu 2003; Ballerstedt et al. 2021; Lokesh et al. 2023; Brown et al. 2023).

However, most microbial processes demonstrated thus far struggle to meet the benchmarks required for economical or environmental viability, even when using energy-rich substrates such as glucose (Xie 2022). The biorefinery approach for waste processing includes other challenges and costs, such as input sorting, purification, and pre-treatment. (Arena and Ardolino 2022). In addition, the biological processing of any waste stream is also complicated by the variability and unpredictability of input feedstocks (Lad et al. 2022; Bergeson et al. 2024).

Strategic nutrient sourcing, taking into account process goals and organismal metabolism and physiology, can help alleviate many of these issues and should be a prime and standard area of investigation (Singh et al. 2017; Noroozi and Jarboe 2023). It is important to keep in mind that these nutrients will not only affect process metrics, but also the economic viability and process footprint (Humbird 2021). While much attention is provided to the cost of the carbon source, the nitrogen component of the media cost is also substantial, thus it is important to choose the correct source and concentration of nitrogen according to specific needs and characteristics of the bioprocess to maximize production (Rajagopalan 2016).

Generally, the available types of nitrogen can be divided into organic and inorganic groups. Microbes can have different preferences for nitrogen source types that vary based on their species, the process at hand, and the metabolic accessibility (Montesinos et al. 1996; Gonçalves et al. 2014; Scott et al. 2021; Noroozi and Jarboe 2023; Opulente et al. 2024). These preferences have been explored previously in the utilization of oil palm sap by *Clostridium acetobutylicum* for the production of biobutanol indicating a preference towards the inorganic nitrogen source ammonium nitrate (Komonkiat and Cheirsilp 2013) and in *Clostridium beijerinckii* indicating preference towards the organic nitrogen source yeast extract (Tekin et al. 2023). The economic impact of strategic nutrient sourcing is demonstrated in the biomanufacturing of penicillin, where the use of the protein- and amino acid-rich corn steep liquor was a key step towards achieving economic viability (Davey and Johnson 1953; Swartz 1979). The use of corn steep liquor as a cheap nutrient source has become industrially commonplace, though it has been noted that there is an insufficient supply to meet the biomanufacturing demand (Humbird et al. 2011).

It is well understood that yeast cells are equipped with complex systems to sense and respond to varying concentrations and sources of nitrogen using a mechanism termed nitrogen catabolite repression (Strathern et al. 1982; Rødkaer and Faergeman 2014). This system prevents cells from diverting resources towards the utilization of non-preferred nitrogen when preferred sources are available (Magasanik and Kaiser 2002). Nitrogen sources are also known to play a key role in cell longevity and fermentation performance (Santos et al. 2016). For *Saccharomyces cerevisiae*, the preferred nitrogen sources are a mixture of amino acids and peptides (Magasanik and Kaiser 2002).

In this study, we will explore the baseline nitrogen preference of a non-conventional yeast *Candida maltosa*. This organism has been previously touted for its uptake capability of thermally oxo-degraded plastic waste (TOD) (Brown et al. 2023) and was subsequently improved through adaptive laboratory evolution (Rodriguez Ocasio et al. 2026). Our exploration will compare preferences during utilization of glucose to preferences during utilization of various hydrophobic substrates, including TOD. We show that using an organic nitrogen source will improve growth on these substrates. We will also investigate some of the cellular-level impacts of these various nitrogen sources and gain insight into the mechanisms and challenges of TOD utilization. The results show an improve in the growth of *C. maltosa* on hydrophobic substrate when an organic nitrogen source is used.

## Material and methods

### Strains and culture conditions

The yeast species used in this study are listed in Table 1. *Candida maltosa* (NRRL Y-17677) was the focal organism. Seed cultures were grown in YPAD rich medium consisting of 10 g/L yeast extract, 20 g/L peptone, 0.04 g/L adenine sulfate and 20 g/L dextrose. Seed cultures were washed twice with sterile nano-pure water to remove any remaining nutrients before being used for inoculation to an OD_600_ of 0.1. All experimental samples were grown in Yeast Nitrogen Base (YNB) defined medium using BD Difco Yeast Nitrogen Base without ammonium sulfate and amino acids supplemented with 20 g/L dextrose. YNB with 0.05 M dextrose was used as a control in all experiments with model compounds.

**Table 1:** Yeast strains used in this study and their ATCC strain designation

Genus and Species	Strain designation (NRRL Y-)
*Candida maltosa*	17677
*Wickerhamomyces subpelliculosus*	1683
*Metschnikowia pulcherrima*	7111
*Saccharomyces cerevisiae*	12632
*Scheffersomyces stipitis*	7124
*Rhodotorula toruloides*	1091
*Kluyveromyces marxianus*	8281
*Yamadazyma philogaea*	7813
*Wickerhamomyces anomalus*	366
*Kazachstania unispora*	1565
*Pichia membranifaciens*	2026
*Candida boidinii*	2332
*Kazachstania exigua*	12640

Nitrogen sources and concentrations used in this study include 5.0 g/L ammonium sulfate (AmSO_4_; Fisher Scientific—CAS 7783–20-2) and 2.0 g/L casamino acids (CAA—CH_1.91_N_0.23_O_0.52_S_0.005_; USBiological Life Sciences—C2080), and 1 g/L algae extract (AE—12.6 wt/wt% nitrogen). The composition of amino acids in casamino acids, according to the manufacturer, is listed in Table S1. All conditions were tested in triplicate. Media without cell inoculation was used as a negative control to ensure the absence of contamination. All cultures were grown at 30 ℃ with 250 rpm orbital shaker (MaxQ 6000). Growth was measured using optical density at 600 nm (OD_600_) and dry cell weight. Dry cell weight measurements were done by washing 1 mL of cell culture twice with sterile nano-pure water and drying at 60 ℃ for a minimum of 5 h.

### Hydrophobic model compounds and TOD

As described previously (Rodriguez-Ocasio et al. 2022), our set of model compounds (Table S2) included saturated hydrophobic compounds from five functional groups (alcohols, alkanes, alkenes, esters, and carboxylic acids) with even carbon chain lengths (14, 18, and 22). As in this prior study, these model compounds were added to the media at a concentration of 0.05 M, where these values were initially selected based on the limited solubility of the substrates in the aqueous media.

Thermally oxo-degraded (TOD) plastic waste was prepared according to the method previously described (Brown et al. 2023) with the feedstock containing 50% wt/wt high-density polyethylene (HDPE) and 50% wt/wt polypropylene (PP) pellets. The thermal oxo-degradation process can produce separate product fractions with varying compositions (Fig. S3). Those recovered at a temperature of 105 ℃, referred to as stage fraction 1 (SF1) have higher boiling points and solidify as they cool to room temperature. Stage fraction 2 (SF2) is passed through a heated in-line filter prior to cooling down and have similar boiling points as SF1. The bulk product of the aforementioned fractions was referred to as wax and used in experiments at 5.0 g/L. The remaining lighter compounds that do not solidify are referred to as liquid hydrocarbons.

### Preparation of algae extract (AE)

*Anabaena* sp. cells were grown in BG-11 medium for 10 days at 25 ℃ under illumination of natural light at 60 μmol·m^−2^·s^−1^. Cells were harvested and dried, then resuspended in a solution of 1:20 vol/vol DI water and 0.1 M Tris–HCl buffer at pH 7.0 four times. Extracted biomolecules were centrifuged at 15,000 × g for 5 min at 4 ℃, after which the supernatant was collected, filtered, and freeze-dried. This extract was added to the media from a concentrated stock of 5.0 g AE/L in nano-pure water. The total nitrogen content of the extract was determined using Dumas’s combustion method according to AOAC method 993.13. The protein percentage was estimated by multiplying by 6.25.

### CHN analysis

CHN elemental analysis was performed by Iowa State University’s Chemical Instrumentation Facility. Elemental analysis results (%C, %H, and %N) were acquired using Thermo FlashSmart combustion analyzer. Acetanilide (Sigma—CAS 103-84-4) was used as the calibration standard. Combustion and reduction temperatures were at 925 ℃ and 640 ℃, respectively. Finally, the precision and accuracy of the measurements were set at ± 0.3% for each element.

### Intracellular protein content

Intracellular protein extraction was carried out using Y-PER™ Yeast Protein Extraction Reagent (Thermo 78990) following the recommended protocol. In summary, cells were harvested and washed using nano-pure water, and approximately 50 mg of wet cell pellet was resuspended in 150 μL of Y-PER reagent and vortexed at 2500 × g at room temperature for 20 min. Cell debris was pelleted by centrifugation at 14,000 × g for 10 min. A second extraction was determined not to be necessary for the improvement of extraction efficiency (data not shown). The lysate (supernatant—100 μL), consisting of intracellular proteins, was isolated and used for protein concentration determination using a NanoDrop at 280 nm (Thermo Scientific NanoDrop 2000). Several sample groups grown in AmSO_4_ did not produce enough biomass for accurate protein measurement. These were particularly observed in longer chain length compounds (alkene C22, alcohol C22, ester C22, and carboxylic acid C18 and C22). These compounds were removed from protein analysis because they did not meet minimal growth thresholds.

For SDS-PAGE analysis, the lysates were adjusted to a protein concentration of 4 mg/mL in phosphate buffer saline (PBS). This was mixed with equal volume of 2X Laemmli Sample Buffer (Bio-Rad—Cat #1610737). Twenty μL of the adjusted solution was loaded into a 10% Bio-Rad precast gel against a 200 kDa protein ladder (Millipore—mPAGE™-MES SDS PAGE). The gels were stained with 50% vol/vol water, 40% vol/vol acetic acid, 10% vol/vol methanol, 10 g/L coomassie blue R-250 for one hour before being de-stained by a mixture of 50% vol/vol water, 40% vol/vol acetic acid and 10% vol/vol methanol overnight.

Selected protein bands were analyzed at the Iowa State University Protein Facility. The desired bands were digested directly from the gel using a trypsin digestion. The samples were then subjected to liquid chromatography and analyzed by MS/MS using the Q Exactive™ Hybrid Quadrupole-Obrbitrap Mass Spectrometer. Raw data was analyzed with Thermo Scientific’s Proteome Discoverer Software using Mascot to identify the spectral data against *Candida maltosa* Xu316 database. Coverage values indicate the percentage of the protein sequence covered by the peptides.

### Fatty acid extraction

Total fatty acid extraction and quantification was performed according to a modified protocol (Bonaventure et al. 2003). In short, cells were harvested and dried in a freeze-drier overnight or until they were completely dry. 10 mg of dry biomass was transferred to a clean glass tube and 20 μL of C19:0 triacylglycerol standard was added directly to the pellet at a final concentration of 2.5 mg/mL. Then, 500 μL of barium hydroxide (Aldrich 433373—100 g/L) was added and the samples were sonicated in a water bath three times for 10 min with 1 min of vortexing at 2500 × g in between. After sonication, 550 μL of 1,4-dioxane (Aldrich 123-91-1) was added and the mixture was heated at 110 ℃ for 24 h in a tightly capped tube. Samples were cooled to room temperature and 6 M HCl was added such that a value less than or equal to 4.0 was observed using pH indicator strips.

Fatty acids were recovered by a two-step hexane extraction. 2 mL of hexane was added to the samples, vortexed for 5 min, centrifuged at 2000 × g for 10 min at 25 ℃, and the hexane layer was transferred to a new tube. The remainder of the sample was re-extracted by repeating this step. These hydrolyzed fatty acids were then methylated by concentrating the samples under nitrogen flow and adding 3 mL of a mixture of methanol: sulfuric acid (98:2 vol/vol) followed by incubation of the samples at 55 ℃ for 30 min. Then, 3 mL of a sodium chloride solution (9 g/L) and 1 mL of hexane were added. The mixture was then vortexed for 5 min at 1,900 rpm, and centrifuged at 2000 × g for 5 min before transferring 750 μL of the organic layer into a new tube for silylation. Samples were dried again under nitrogen flow and 100 μL of N, O-bis(trimethylsilyl)trifluoroacetamide (BSTFA) with 1% vol/vol trimethylchlorosilane (TCMS) was added, followed by 2 min of vortexing. Samples were incubated for 30 min at 60 ℃ and diluted with hexane to the desired volume.

Derivatized samples were analyzed by GC–MS at the Iowa State University W.M. Keck Metabolomics Research Laboratory (RRID:SCR_017911). GC–MS analyses were performed with an Agilent 6890 gas chromatograph coupled to a model 5973 Mass Selective Detector (Agilent Technologies, Santa Clara, CA). The column used was HP-5MS 5% Phenyl Methyl Silox with 30 m × 250 µM × 0.25 µm film thickness (Agilent Technologies). One microliter of sample was injected with the inlet operating in splitless mode and held at a constant temperature of 280 °C. The oven temperature was programmed as follows: an initial temperature of 70 °C was increased to 100 °C at 5 °C/min and held for 2 min before being further increased at 17 °C/min to a final temperature of 320 °C, which was held for 8 min. Helium was used as a carrier gas at a flow rate of 1 mL/min. The MS transfer line was held at 280 °C. Mass Spectrometry detection was performed using electron ionization at 70 eV and source temperature and quadrupole temperature were set at 230 °C and 150 °C, respectively. The mass data were collected in the range from m/z 40 to m/z 800.

Identification and quantification were conducted using AMDIS (Automated Mass spectral Deconvolution and Identification System, National Institute of Standards and Technology (Gaithersburg, MD) with a manually curated retention indexed GC–MS library with additional identification performed using the NIST17 and Wiley 11 GC–MS spectral library (Agilent Technologies, Santa Clara, CA). Final quantification was calculated by integrating the corresponding peak areas relative to the area of the internal standards. Raw data were normalized to the amount of tissue used. Statistical evaluation of the non-targeted GC–MS data was conducted with the R-based statistical package MetaboAnalyst (Pang et al. 2021). Important molecular features were elucidated using MetaboAnalyst’s multivariate analysis tools.

### Cell surface hydrophobicity and membrane polarization measurements

The microbial cell surface hydrophobicity was measured using the method described by Rosenberg et al. (1980). In short, the yeast cells grown on various conditions for 48 h were pelleted and washed twice with PBS pH 7.0 at 1500 × g for 5 min. The samples were adjusted with the PBS to an OD_600_ of 0.4 (A_0_) in 1.2 mL. Next, 300 μL of hexadecane was added to the microbial suspension and samples were vortexed vigorously at 2500 rpm for 120 s. Samples were then held at room temperature for 10 min, allowing for separation of the organic and aqueous layers. 1 mL of the aqueous phase was removed and transferred into a cuvette. Absorbance was measured again at an OD_600_ (A_1_). Cell surface hydrophobicity percentage was estimated using Eq. 1 (Chrzanowski et al. 2008).

1 $$ Cell\,Surface\,Hydrophobicity\,\left( \% \right) = \left( {\frac{{A_{0} - A_{1} }}{{A_{0} }}} \right) \times 100 $$

1,6-Diphenyl-1,3,4-hexatriene (DPH) was used as a probe to assess the structural and dynamical features of the lipid bilayer (Mykytczuk et al. 2007; Poojari et al. 2019). First, protoplasts were prepared using a modified protocol by Schwencke and Nagy (Schwencke and Nagy 1978). In summary, we grew the cells to an OD_600_ of at least 2.0. The OD_600_ was adjusted to 2.0 in 10 mL of sterile nano-pure water as needed. Then the cells were pelleted by centrifugation at 1,500 × g for 5 min. The cell pellet was resuspended in 1 M sorbitol, 25 mM ethylenediaminetetraacetic acid (EDTA), 50 mM dithiothreitol (DTT), pH 8.0 and pelleted again. This pellet was resuspended in 1 M sorbitol (solution 2), followed by another pelleting. This final pellet was resuspended in 1 M sorbitol, 1 mM EDTA, 10 mM sodium citrate buffer, pH 5.8. To determine the efficiency of the protoplasting, a baseline OD_800_ is measured using 200 μL of washed cells in 2 mL of solution 2 and 800 μL of 50 g/L sodium dodecyl sulfate (SDS) (O_0_). Afterwards, 15 μL of zymolyase solution in nano-pure water (60 U/mL) was added and the mixture was incubated for 1 h at 30 ℃ and 200 rpm. A second measurement of OD_800_ was collected after adding 200 μL of the zymolyase treated cells to 2 mL of solution 2 and 800 μL of 50 g/L SDS (O_1_). The efficiency is measured using Eq. 2 until its value reaches 90% or above. The suspension was then centrifuged at 1,500 × g for 5 min and resuspended in solution 2 at the desired final working concentration (Lyu et al. 2021).

2 $$ {{\% }}Efficiency = \left( {\frac{{O_{0} - O_{1} }}{{O_{0} }}} \right) \times 100 $$

The prepared protoplasts were then prepared for measurement of DPH polarization, using a modified protocol by Liu et al. (Liu et al. 2013). DPH was prepared in a stock solution of N, N-dimethylformamide. The protoplasts were separated into two tubes of a working sample and a no-DPH control. DPH was added to the samples at a final concentration of 0.4 μM. The samples were then aliquoted into black-bottomed Nunclon Delta Surface 96-well plates. The Synergy Multi-Mode microplate reader from Bio Tek was used to measure polarization with 360/40 nm for excitation in a vertical position and 460/40 nm filter for emissions in the vertical (I_VV_) or the horizontal (I_VH_) position over 15 min. The plate reader temperature was maintained at 30 ℃. Polarization was calculated using Eq. 3 (Pandey and Mishra 1999), where G is the grating factor specific to the instrument, defined by the ratio of I_VV_ and I_VH_. This value was calculated previously for our instrument and was considered constant at 1.102 (Santoscoy and Jarboe 2021)

3 $$ Polarization{ }\left( P \right) = \frac{{I_{VV} - GI_{VH} }}{{I_{VV} + GI_{VH} }} $$

### Statistics

Microsoft Excel was used for statistical analysis. Unpaired *t*-tests were performed with a maximum significance level of 0.05 using GraphPad. Only differences with a *p-*value of 0.05 or less were marked with significance. Total uncertainty for calculated results was determined from the propagation of error based on individual measurements of standard deviation.

## Results and discussion

In this study, we evaluated the implications of using different nitrogen sources, namely ammonium sulfate (AmSO_4_—inorganic), casamino acids (CAA—organic) and algae extract (AE—organic) in growth with hydrophobic model compounds and thermally oxo-degraded plastic waste (TOD). The assessment of nitrogen preferences in bioprocesses provides valuable information for the control and improvement of process efficiency and economic viability. This strategy can also help avoid toxic by-products that could lead to premature growth termination (Zhang et al. 2018).

*C. maltosa* has the apparent ability to utilize a broader spectrum of hydrophobic model compounds relative to other yeast species (Rodriguez-Ocasio et al. 2022), making it an attractive microbial platform for the upcycling of hydrocarbon-rich wastes. This selection of a platform organism based on carbon source utilization patterns is common (Sitepu et al. 2014; Zhang et al. 2021; Wan et al. 2023). However, consideration and optimization of nitrogen source and concentration can pave the way to operation at industrial scale (Noroozi and Jarboe 2023). In this study, we demonstrate that strategic nitrogen provisioning impacts process metrics across a range of simple and complex carbon sources, including hydrophobic mixtures of hydrocarbons.

**Fig. 1:**
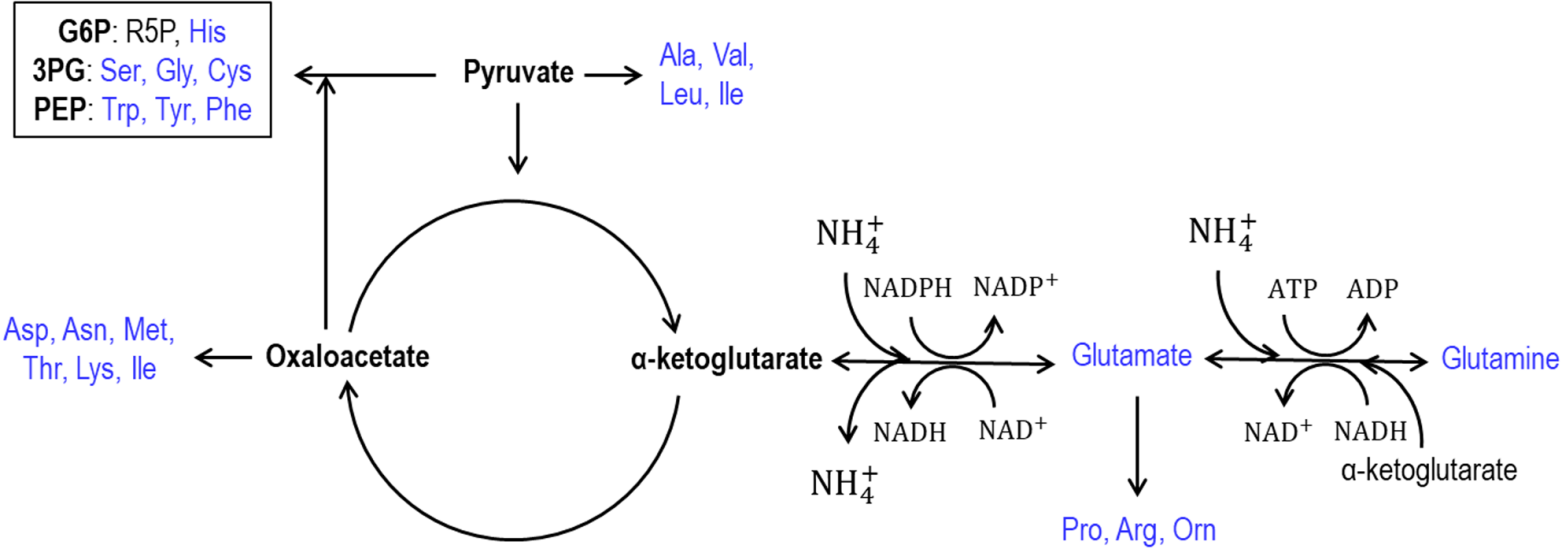
Structure of pathways for nitrogen assimilation to amino acids. Figure adapted from Magasanik, 2003. G6P, glucose 6-phosphate; R5P, ribose 5-phosphate; 3PG, 3-phosphoglycerate; PEP, phosphoenolpyruvate

The main pathway for inorganic nitrogen assimilation leads to the formation of glutamate and glutamine, which are then used as nitrogen donors for the production of virtually all other biomolecules (Fig. 1) (Ikeda et al. 1996). The nitrogen source preference during glucose utilization has been extensively studied for industrially relevant microorganisms such as *Escherichia coli* (Reitzer 2003) and *Saccharomyces cerevisiae* (Magasanik and Kaiser 2002). In this scenario, ammonia is generally preferred relative to amino acids, based on growth characteristics (Wang et al. 2016). However, this preference can differ depending on the carbon source provided (Zhang et al. 2018). To the best of our knowledge, *C. maltosa*’s general and specific nitrogen source preference has not been studied.

### Nitrogen type impacts the elemental composition of cells

Awareness of the aforementioned nitrogen preferences is important in deciding which nutrients to incorporate into the fermentation media. Changes in the nitrogen source have been shown to change the elemental nitrogen, carbon, and oxygen content of the cells (Folsom and Carlson 2015; Ansari et al. 2025). Our CHN analysis of *C. maltosa* shows its elemental composition is similar to *S. cerevisiae* when both are grown on inorganic nitrogen (Table 2). However, *C. maltosa*’s relative abundance of nitrogen decreases by roughly 30% when organic nitrogen is used. This highlights the importance of using biomass elemental analysis to inform the media composition. For example, in order to convert glucose to 5 g/L of biomass, a biomass composition of C_1_N_0.13_H_1.9_O_0.9_ requires 0.31 g N/L, but a composition of C_1_N_0.09_H_1.8_O_1_ requires only 0.20 g N/L.

**Table 2:** CHN analysis of *C. maltosa* biomass grown in YNB and glucose with ammonium sulfate or casamino acids. Values in the parenthesis indicate the standard deviation.

Carbon source	C mass fraction (g/g biomass)	Nitrogen source	N mass fraction (g/g biomass)
*C. maltosa* (this study)			
Glucose	0.41^a^ (0.01)	AmSO_4_	0.062^a^ (0.002)
	0.393^a^ (0.005)	CAA	0.0398^b^ (0.0007)
TOD	0.462^b^ (0.004)	AmSO_4_	0.077^c^ (0.003)
	0.452^b^ (0.011)	CAA	0.081^c^ (0.005)
*S. cerevisiae* (Duboc et al. 1995)			
Glucose	0.4243 (0.0004)	Ammonia ions	0.0641 (0.0003)

Statistical analysis indicated for the comparison of *C. maltosa* samples only. Superscript letters indicate statistically significant (*p* < 0.05 ) differences for *C. maltosa* within a single element. Values with the same letters are not significantly different

Two of the most commonly used media for yeast are Yeast Nitrogen Base (YNB) and YPAD (yeast extract, peptone, adenine sulfate, and dextrose). As a defined medium, YNB contains 1.06 g N/L in the form of 5 g/L ammonium sulfate (AmSO_4_). Contrastingly, because of variability in the composition of the rich media components, YPAD provides 4.2 ± 0.5 g N/L, mainly in the form of amino acids and nucleotides. We used YNB as the basis of our media and incorporated changes in the carbon (hydrophobic model compounds and TOD) and nitrogen (algae extract and casamino acids). These conditions were tested against YNB with standard carbon (glucose) and nitrogen (AmSO_4_) sources.

### Algae extract (AE) is a preferred nitrogen source on hydrophobic substrates

One of the nitrogen sources explored here is hydrolyzed cyanobacteria *Anabaena sp*. This hydrolysate consists of 65% wt/wt of amino acids, 18% wt/wt carbohydrates, and 3% wt/wt lipids (Dunn and Wolk 1970). The freeze-dried hydrolysate used here contained 79% wt/wt proteins and a bulk nitrogen content of 12.6% wt/wt. *Anabaena sp*. is grown via the fixation of inorganic nitrogen from air and subsequent storage of organic nitrogen in the form of biomolecules (Clares et al. 2014). A similar scenario was investigated by Kightlinger et al. (2014) in which *Escherichia coli* and *S. cerevisiae* biomass production on algae extract was comparable to the use of commercial yeast extract.

**Fig. 2:**
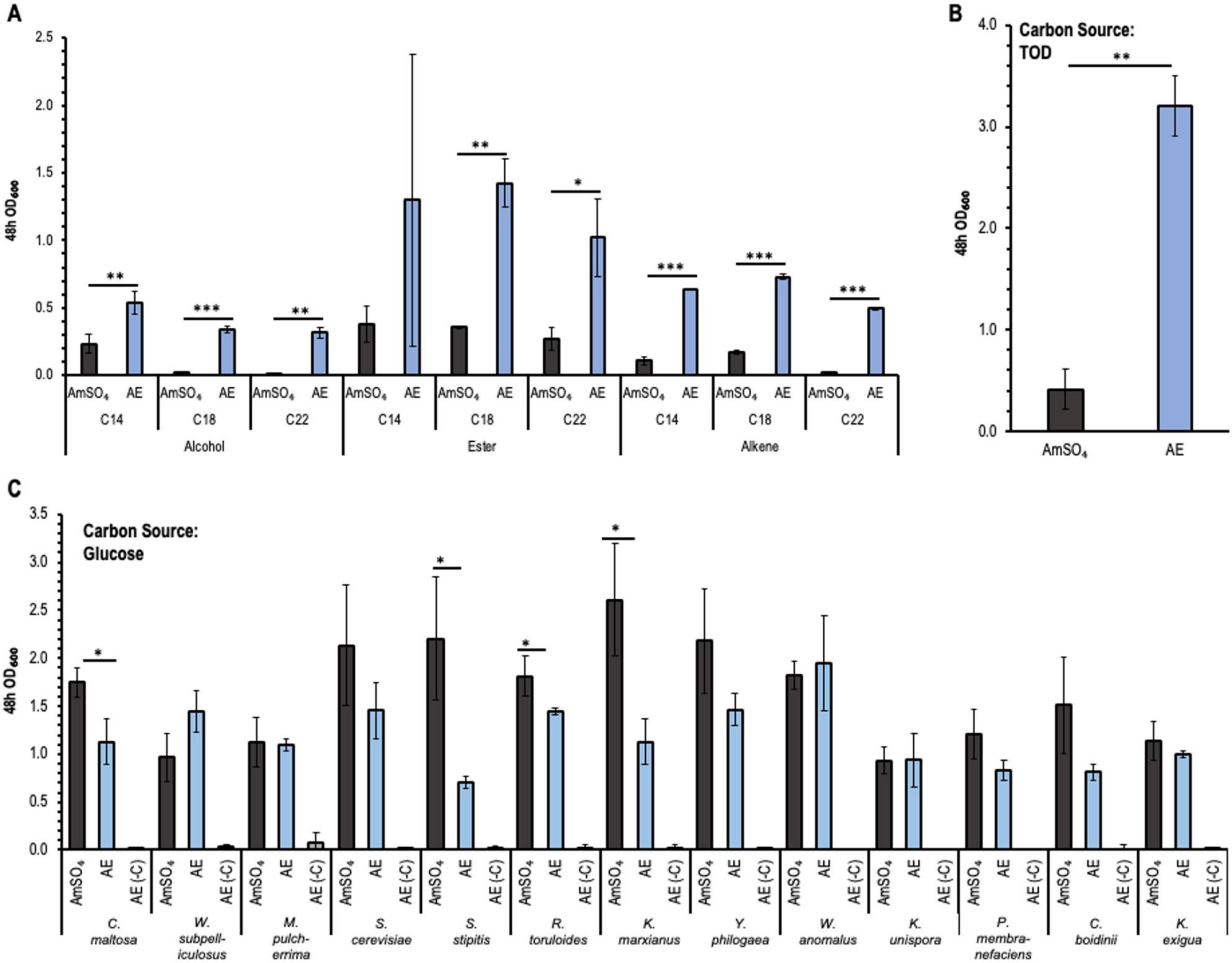
Incorporation of 1 g/L *Anabaena* sp. extract (AE) in the uptake of hydrophobic carbon substrates (model compounds) and TOD results in a boost in growth. **A** 48-h growth indicated by observed OD_600_ of *C. maltosa* in hydrophobic hydrocarbon as the carbon source and AE as the nitrogen source. **B** Growth of *C. maltosa* on TOD (50% wt/wt HDPE, 50% wt/wt PP) with AE as the nitrogen source compared to ammonium sulfate. **C** Growth of 13 yeast species using AE as the nitrogen source compared to ammonium sulfate in glucose as the carbon source. A no-carbon control (AE(-C)) was included to show that AE is not being utilized as the carbon source. All experiments were conducted at 30̊C and 250 rpm in YNB. Three biological replicates were used for all conditions. The significant changes relative to the control condition are calculated using an unpaired t test and are indicated as follows: *, *p* < 0.05; **, *p* < 0.001; ***, *p* < 0.0001

AE served as the nitrogen component of YNB media with glucose at concentrations ranging from 0.5 to 3 g AE/L (Fig. S1). A concentration of 1 g AE/L (0.12 g N/L) was determined to be optimal in terms of biomass production, with further increases inhibiting *C. maltosa* growth. When hydrophobic model compounds were used as carbon source, the biomass production by *C. maltosa* was significantly higher with AE as nitrogen source relative to AmSO_4_ (1.06 g N/L) (Fig. 2A). This was especially noteworthy for alcohols and alkenes with longer chain lengths, which showed little to no growth on AmSO_4_. These compounds are typically observed in thermal oxo-degradation of polyethylene but have a non-inhibitory nature (Guzik et al. 2014; Rodriguez-Ocasio et al. 2022). The finding that use of a different nitrogen type improves utilization of such molecules despite the lower nitrogen dosing, provides motivation for using organic nitrogen in fermentations. Similar results were observed when TOD was used as a carbon source (Fig. 2B), with more than a sixfold increase in the observed biomass concentration at 48 h. Note that no growth was observed in YNB media containing 1 g AE/L in the absence of a distinct carbon source (Fig. 2C).

All of the 13 yeast species tested here were able to utilize AE as nitrogen source, with glucose as the carbon source (Fig. 2C). While we observed that organic nitrogen sourcing increased *C. maltosa* biomass production from hydrophobic compounds and from TOD, the opposite trend was observed when glucose was carbon source. Specifically, biomass production was significantly decreased relative to inorganic nitrogen (Fig. 2C). Similar results were observed for *S. stipites, R. toruloides* and* K. marxianus*. The other yeast species showed similar growth on both types of nitrogen (Fig. 2C). This preference for inorganic nitrogen during glucose utilization is consistent with prior studies (Wang et al. 2016).

### Casamino acids (CAA) is a comparable organic nitrogen source to AE

AE shows potential for use in the fermentation of complex hydrophobic substrates (Fig. 2B). However, the complex and variable nutrient profile of AE (Fig. S2) can confound our understanding of the system and the underlying mechanisms of changes. Therefore, we selected CAA as a model amino acid-based nitrogen source. Specifically, 2 g CAA/L was used to provide roughly 0.13 g N/L.

**Fig. 3:**
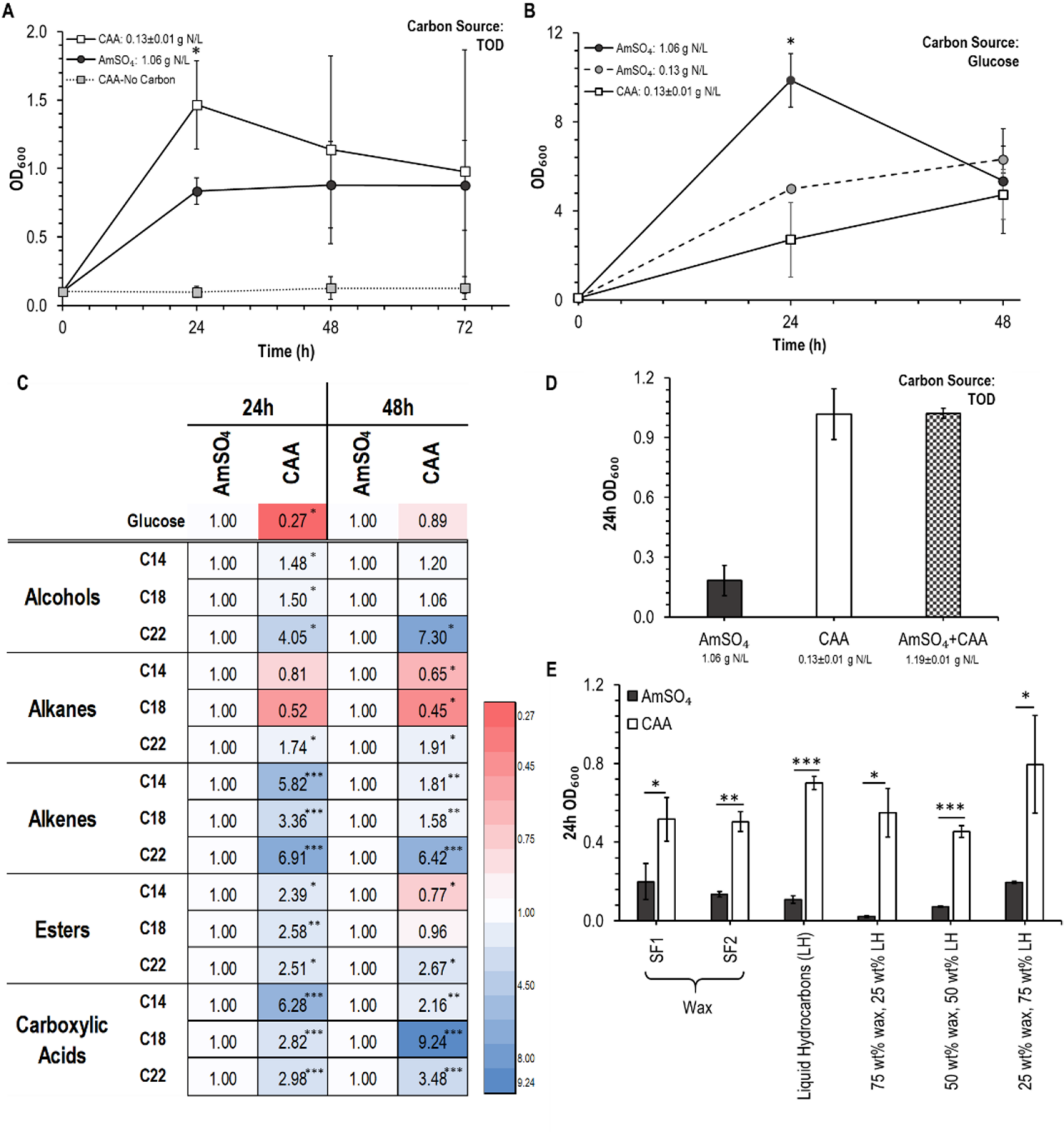
Casamino acids used as the nitrogen source in YNB media increase the growth of C. maltosa when model compounds are used as the carbon source. **A** Use of 2 g/L of casamino acids (CAA) as nitrogen source in comparison to 5 g/L of ammonium sulfate (AmSO_4_) when 5 g/L thermally oxo-degraded (TOD) plastic waste (50% wt/wt HDPE and 50% wt/wt PP; insoluble) was used as the sole carbon source. A no-carbon control (CAA-No carbon) is included without TOD to ensure that CAA is not being used as a carbon source. **B** Growth comparison of AmSO_4_ and CAA in glucose. **C** 24- and 48-h growth of C. maltosa in 0.05 M model compounds of chain length 14 (C14), 18 (C18), and 22 (C22) with 5 g/L of AmSO_4_ or 2 g/L of CAA normalized to the growth in AmSO_4_. **D** C. maltosa’s growth with AmSO_4_, CAA and both. **E** *C. maltosa’s* growth with CAA compared to AmSO_4_ in various fractions of the TOD as carbon sources. The significant changes relative to the control condition are calculated using an unpaired t-test and are indicated as follows: *, *p* < 0.05; **, *p* < 0.001; ***, *p* < 0.0001

Similar to the observations with AE, this provisioning of CAA increased growth on TOD compared to AmSO_4_ (Fig. 3A). However, this difference was only significant at 24 h. Again, no growth was observed when CAA were provided in the absence of a distinct carbon source. Dry cell weight measurements of *C. maltosa* growth on TOD support the conclusion that growth is increased with CAA relative to AmSO_4_ (Table S3). Similarly, utilization of glucose as carbon source was associated with a preference for inorganic nitrogen (Fig. 3B). To account for the difference in total N dosing, we compared growth on glucose with 0.13 g N/L provided as either organic (CAA) or inorganic (AmSO_4_) (Fig. 3B). Growth was similar in both conditions.

When model hydrophobic compounds were used, growth trends on CAA and AmSO_4_ closely represented those of AE (Fig. 3C). Specifically, the use of organic nitrogen was associated with a significant increase in biomass production for almost all model compounds. This effect was more pronounced in the first 24 h of the 48-h growth. Notably, compounds with higher chain lengths consistently grew better on these model compounds with CAA as nitrogen source over the entire growth period (Fig. 3C).

Furthermore, we investigated if the increase in growth on TOD at 24 h was due to the type of nitrogen source or the nitrogen dose by providing both 5 g/L of AmSO_4_ and 2 g/L of CAA simultaneously, for a total dose of 1.19 g N/L (Fig. 3D). No further increase in biomass production was observed compared to those containing only 2 g CAA/L. This indicates that AmSO_4_ is not the optimal or necessary choice of nitrogen in this fermentation, despite the lower amount of nitrogen provided by the CAA.

### *C. maltosa* growth with CAA is robust across different TOD fractions

The thermal oxo-degradation process converts plastic to a complex mixture of hydrocarbons that can be collected in distinct fractions (Brown et al. 2023). The composition and relative distribution of these fractions are subject to batch-to-batch variability in response to perturbations in the feed composition and operational parameters, similar to the challenges associated with utilization of lignocellulosic biomass (Jarboe et al. 2022). There is an inherent conflict between the desire to utilize all of the carbon exiting the TOD process and the desire to provide the microbial biocatalyst with a substrate that supports the highest amount of carbon utilization. Therefore, it is important to evaluate the microbial system’s growth on these fractions. Similar to other complex feed streams, reliability of the microbial behavior is of the utmost value (Lad et al. 2021) and it is important to be equipped with strategies to mitigate these uncertainties. Nitrogen provisioning with organic waste has been used previously to overcome such challenges (Tan et al. 2020; Kot et al. 2020).

The results shown in Figs. 2B and 3A utilized a TOD product that was a combination of the waxy fractions SF1 and SF2 (Fig. S3) and did not include any of the liquid hydrocarbon fraction. *C. maltosa* growth was observed on each of these three individual fractions (Fig. 3E), with each fraction supporting higher growth using organic nitrogen relative to inorganic. Similar trends were observed when the liquid and wax product streams were combined. Thus, the beneficial effect of organic nitrogen sourcing is conserved across the range of TOD products.

### Different nitrogen sources impact media pH differently

Changes in the nitrogen source provided to the media can impact the pH of the growth environment. The optimal pH for yeast species has been reported to be between 4 to 6 depending on culture conditions and the species (Narendranath and Power 2005), and it is well understood that the use of AmSO_4_ leads to the acidification of the media. Therefore, we looked at the changes in the media pH during TOD fermentation to evaluate the impacts of nitrogen source on this variable (Fig. S4). The starting pH for cultures prepared with AmSO_4_ or CAA were similarly measured at 5.5 ± 0.5. However, as growth continued throughout 72 h, it was observed that the cultures supplemented with AmSO_4_ experienced a decline in pH to approximately 3.5 ± 0.5, while the CAA-supplemented cultures maintained a stable pH round 5.5. While the higher pH maintained the latter can promote more growth in the cells, it is also observed that it does not lead to a sustained increase after 24 h, indicating that the better growth in this case is impacted by the pH, but not solely because of it.

The effect of pH of cellular stability and health is well studied; cells need to maintain a constant intracellular pH to allow the function of enzymes and metabolism. This strict monitoring of pH by cells is regulated by pumping out ions which in turn changes extracellular pH (Thomas et al. 2002). This imbalance of intra- and extracellular pH in the case of AmSO_4_ can cause the cells lose the ability to maintain a stable intracellular pH and lead to cell death or dormancy. However, the data gathered in this study suggest that the cease in growth is prompted by nutrient limiting conditions rather than environmental changes.

### Carbon backbone of CAA is a contributor to the increased growth in TOD

Considering the general preference of *C. maltosa* towards glucose relative to individual model compounds (Rodriguez-Ocasio et al. 2022), the provisioning of only these hydrophobic substrates could possibly be categorized as carbon limitation. Hence, the observed increase in growth with CAA is consistent with current understanding of nitrogen assimilation in *S. cerevisiae*, as summarized below.

Production of microbial biomass requires the synthesis or provisioning of amino acids as the building blocks of biological machinery. These amino acids are produced from carbon skeletons distributed throughout central metabolism. The assimilation of inorganic nitrogen requires the carbon skeleton α-ketoglutarate and its amination to the amino acid L-glutamate. Some essential biomolecules are derived from glutamate but also L-glutamate is converted to the amino acid L-glutamine in a second amination reaction. Glutamine then serves as the amino donor for the production of other amino acids. Thus, all inorganic nitrogen enters biosynthesis via the production of glutamate. The amination of α-ketoglutarate requires NADPH and the amination of glutamate requires ATP. Therefore, while CAA cannot serve as sole carbon source (Fig. 3A), its use as nitrogen source not only reduces the need for these aminations, it also decreases the need for routing of carbon to the various amino acid precursors.

This decreased carbon burden is demonstrated through an elemental balance of TOD utilization with these two nitrogen sources. TOD is 83 wt% carbon (Brown et al. 2023) and if the nitrogen source is AmSO_4_, TOD is the only carbon source. In order to synthesize 1 g of biomass containing 46 wt% carbon (Table 2), the cells must metabolize 0.55 g of TOD. Note that these calculations ignore possible uptake or release of CO_2_. Similarly, the biomass contains 6.2 wt% nitrogen that is obtained through the utilization of 0.36 g of AmSO_4_ per g of biomass. These balances become more complicated when the nitrogen source is CAA, because the CAA consists not just of nitrogen (~ 13 wt%), but also 47 wt% carbon. The production of 1 g of biomass containing 8.1 wt% nitrogen requires the consumption of 0.62 g CAA. This consumption brings 0.15 g of carbon, meaning that only 0.19 g of TOD is required per g of biomass. Thus 65% of the carbon converted to biomass can be attributed to CAA. However, the carbon provided by CAA does not appear to solely account for the observed increase in growth associated with the use of CAA (Fig. 3D). Specifically, we observed a 4.6-fold increase in biomass production during growth on TOD when cells were provided with CAA relative to AmSO_4_ (Fig. 3D). This disproportionate increase supports the energetic savings associated with the provisioning of organic nitrogen.

### Utilization of TOD and model compounds requires alternate proteins

As described above, growth on less-preferred molecules, such as the model compounds characterized here, could require more enzymatic steps than preferred molecules, such as glucose (Fickers et al. 2005). The complexity of funneling carbon through a larger network of enzymatic reactions is reminiscent of the complexity of simultaneous utilization of hexose and pentose sugars and of lignin monomers (Ragauskas et al. 2014; Jarboe et al. 2022). Similarly, we hypothesize that the funneling of TOD, a complex, hydrophobic substrate with potentially inhibitory compounds (Gill and Ratledge 1972), into central metabolism for repurposing as microbial biomass is expected to require more enzymatic steps relative to a preferred substrate, such as glucose.

These pathways are expected to be inactive in the absence of specific carbon sources due to costly energetic demands in their production, possibly regulated by the carbon catabolite repression system. For example, *R. toruloides* is known to activate two cellobiose transporters only when the Cbr1 transcription factor is activated by cellobiose (Reyes-Chavez et al. 2026). Characterization of transcriptional regulation of *n*-alkane assimilation in *Y. lipolytica* similarly showed that expression of the Alk proteins is increased in the presence of *n*-alkanes (Fukuda 2013).

The utilization of any substrate requires the transportation of molecules into the cells. While there are various mechanisms for this transport, such as biosurfactant secretion (Hua et al. 2003) and protrusion formation (Fickers et al. 2005; Zvonarev et al. 2017), it is likely that proteins play a role (Claus et al. 2019). For example, for the transport of many hydrophobic drugs (Lanthaler et al. 2011) and lipid molecules (Wong et al. 2019; Balla et al. 2020) into yeast were concluded to be carrier-mediated. The Snq2p, Pdr10p and Pdr5p efflux pumps are involved in alkane export in *S. cerevisiae* (Nishida et al. 2013; Ling et al. 2013). Molecules often require facilitation not only for entering the cell but also for intracellular trafficking (Wong et al. 2019). Thus, it is expected that cells are required to produce more protein in order to utilize these less- preferred compounds relative to glucose.

**Fig. 4:**
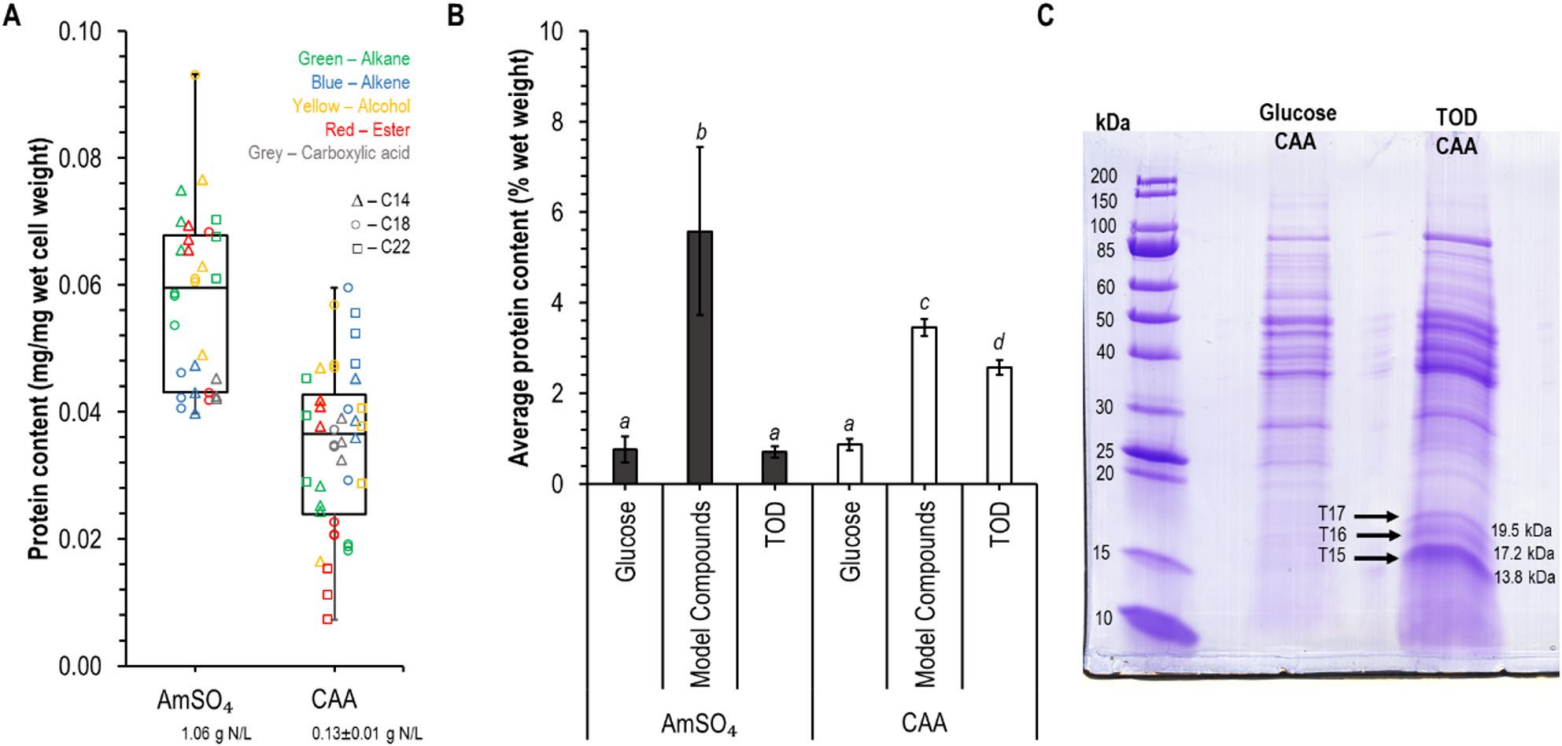
Intracellular protein content of C. maltosa grown on model compounds is variable AmSO₄ or CAA. **A** *C. maltosa’s* protein content (wet weight basis) in model compounds. **B** *C. maltosa’s* average protein content (% wet weight) in glucose, model compounds and TOD when inorganic and organic nitrogen sources are used. Columns marked with different letters are significantly different (*p*-value < 0.05) **C ** SDS-PAGE gel of *C. maltosa’s* protein extract grown on glucose and TOD with CAA as the nitrogen source. Arrows indicate bands subjected to LC–MS

The expectation of a higher protein demand is consistent with the higher relative abundance of proteins observed during use of model compounds relative to glucose, regardless of nitrogen type (Fig. 4B). However, this trend is not wholly conserved when comparing TOD utilization to glucose. While it was observed that protein content on TOD is higher than on glucose when CAA are provided as nitrogen source, no difference between these two carbon sources was detected with provisioning of AmSO_4_. The relatively low protein content with AmSO_4_ could possibly be due to an inability to meet the energetic demand associated with production of these proteins while also meeting the energetic demand of nitrogen assimilation and amino acid biosynthesis.

The boost in growth observed when providing CAA as the nitrogen source warrants a closer look into the cellular implications of such changes in the media. Protein is the most abundant nitrogen-containing biomolecule, and on glucose it accounted for 0.8–0.9 wt% of the wet biomass for both nitrogen sources (Fig. 4A). However, due to the differences in biomass production, the bulk concentrations of intracellular protein were 1.4 ± 0.9 g/L with AmSO_4_ and 0.5 ± 0.3 g/L with CAA (data not shown). Assuming that this protein contains 16 wt% N, this represents 21% conversion of the supplied inorganic nitrogen to protein and 33% conversion of the organic nitrogen, consistent with the relative nitrogen dosing in the two conditions.

We then measured the protein content of cells grown on CAA and AmSO_4_ with each of the 15 model hydrophobic model compounds (Fig. 4B). Within the composite dataset, the average relative protein content was significantly lower for samples grown on CAA (3.5 ± 0.2 wt%, wet basis) relative to AmSO_4_ (5.6 ± 1.8) (p-value 0.046). Thus, while the type of nitrogen source did not impact protein abundance during growth on glucose, a difference was observed during growth on the model compounds. Supplying an organic source of nitrogen provides cells with the building blocks needed for protein synthesis, presumably decreasing the burden of expressing the machinery required for de novo synthesis of these building blocks. The higher protein content observed with AmSO_4_ strengthens the hypothesis that cells experience an increased demand of machinery needed for amino acid biosynthesis in the presence of hydrophobic compounds.

Contrastingly, during utilization of TOD, cells provided with inorganic nitrogen had a lower protein content than cells provided with CAA (Fig. 4B). Comparison of this data to the utilization of glucose shows that for AmSO_4_, the protein content for the two carbon sources is similar, but with CAA the TOD-grown cells had a higher protein content. This lower protein content in cells grown on TOD than on model compounds can possibly be associated with the composition of the TOD substrate. While TOD composition has not been fully quantified, the average molecular weight of 65,000 g/mol indicates the presence of molecules that are substantially larger than our set of model compounds, all of which have a molecular weight below 400 g/mol. Note that when grown on inorganic nitrogen, several of the higher chain length compounds (alkene, alcohol, ester, and carboxylic acid) did not support enough biomass production in 48 h for protein extraction and quantification (Fig. 4A).

This significant increase in the protein content of cells CAA-grown cells in glucose and TOD can also be observed by looking at the elemental analysis of cells in these conditions (Table 1). While CHN results show that the nitrogen content of TOD-grown *C. maltosa* compared to glucose-grown cells significantly changes regardless of the nitrogen, the nitrogen shows a 103% increase for CAA-grown cells between the two carbon sources. This increase is only 24% for AmSO_4_.

### Small molecular-weight proteins are potentially involved in TOD utilization

We performed a high-level proteomic survey of cells supplied with organic nitrogen during growth on glucose and on TOD. Three low-molecular weight proteins were prominent during utilization of TOD but not during utilization of glucose. These three proteins were identified by LC–MS (Fig. 4C) and listed in Table 3.

**Table 3:** Proteins identified using LC–MS

Code	Detected protein	UniProt Acc #	Coverage (%)	MW (kDa)
T17	Glycine zipper 2TM domain-containing protein	M3K6K7	59	19.5
T16	Adenosine 5′-monophosphoramidase HNT1	M3J1B7	61	17.2
T15	Oleate-induced peroxisomal protein POX18	M3JTI3	91	13.8

T15 is the nonspecific lipid transfer protein POX18 (Szabo et al. 1989; Tan et al. 1990), found mainly in peroxisomes and known to be induced by the C18:1 fatty acid oleate (Kamiryo and Okazaki 1984). The abundance of peroxisomes is known to increase when *Candida tropicalis* utilizes mixtures of C10-C13 alkanes (Osumi et al. 1974) and we have visualized an increased abundance of intracellular compartments during growth of *C. maltosa* on TOD-HDPE relative to glucose (Rodriguez-Ocasio, in preparation). This production of peroxisomes is linked to fatty acid oxidation (Susumu Kawamoto et al. 1978; Tan et al. 1990), a vital mechanism for production of ATP in the absence of glucose (Fukui and Tanaka 1979; Cahill 2006). It is also known that oleic acid (C18:1 fatty acid) stimulates fatty acid oxidation activity in murine muscle cells (Lim et al. 2013). *S. cerevisiae* cells unable to form peroxisomes cannot utilize oleate as sole carbon source due to their inability to funnel fatty acids into central carbon metabolism (Erdmann et al. 1989). Therefore, fatty acid oxidation, dependent upon POX18 and peroxisomes, appears to possibly play a role in the utilization of TOD in *C. maltosa*.

T17 is the glycine zipper 2 transmembrane (TM) domain-containing protein (Table 3). This type of lipoprotein motif is relatively common and has been shown to have a substantial effect on membrane structure and physiology (Janssens et al. 2024). Such proteins can exist in multiple oligomerization states, with glycine being the most common interfacial residue, hence the name glycine zipper (Kim et al. 2005). These motifs are considered to be a hallmark of Alzheimer’s and Prion disease, exemplified by the presence of fibrillar deposits on the surface of brain cells (Aguzzi and Haass 2003). These deposits are thought to be cation channels (Kourie and Culverson 2000) that might play a role in neural cell death due to disruption of ion homeostasis (Lin et al. 2001).

Images of these formations on the surface of mouse neuroblastoma cells (Lin et al. 2001) strike a close similarity with ‘canals’ observed on the surface of *C. maltosa* during growth on hexadecane with AmSO_4_ (Zvonarev et al. 2017; Rodriguez-Ocasio et al. 2026). These canals are known to be associated with polyphosphates, which act as protein scaffolding agents, and are induced under nutrient limitation and oxidative stress (Ault-Riché et al. 1998; Guan and Jakob 2024). Although TOD is carbon rich, it consists of a variety of compounds with low solubility and less energy-producing potential, which may lead to a condition of nutrient limitation. Thus, this particular transmembrane protein may be involved in the utilization of these hydrophobic compounds.

Finally, T16 is histidine triad nucleotide binding protein HNT1. Its activity as an adenosine 5’-monophosphoramidase has been observed (Fankhauser et al. 1981; Bieganowski et al. 2002). Adenosine 5′-monophosphoramidate is derived from an organosulfur biosynthesis intermediate and HNT1 converts adenosine 5′-monophosphoramidate to adenosine monophosphate and ammonia, though it is suspected that it has activity on other biomolecules (Fankhauser et al. 1981; Bieganowski et al. 2002). Thus, this enzyme may somehow be associated with the phosphate-rich molecules detected with *C. maltosa*’s cell wall canals. Some members of this superfamily, namely the Fhit, function as a tumor suppressor protein in humans (Siprashvili et al. 1997) and exhibit diadenosine polyphosphate hydrolase activity (Brenner 2002).

### Cell membrane properties reflect modifications of fatty acid content and composition

**Fig. 5:**
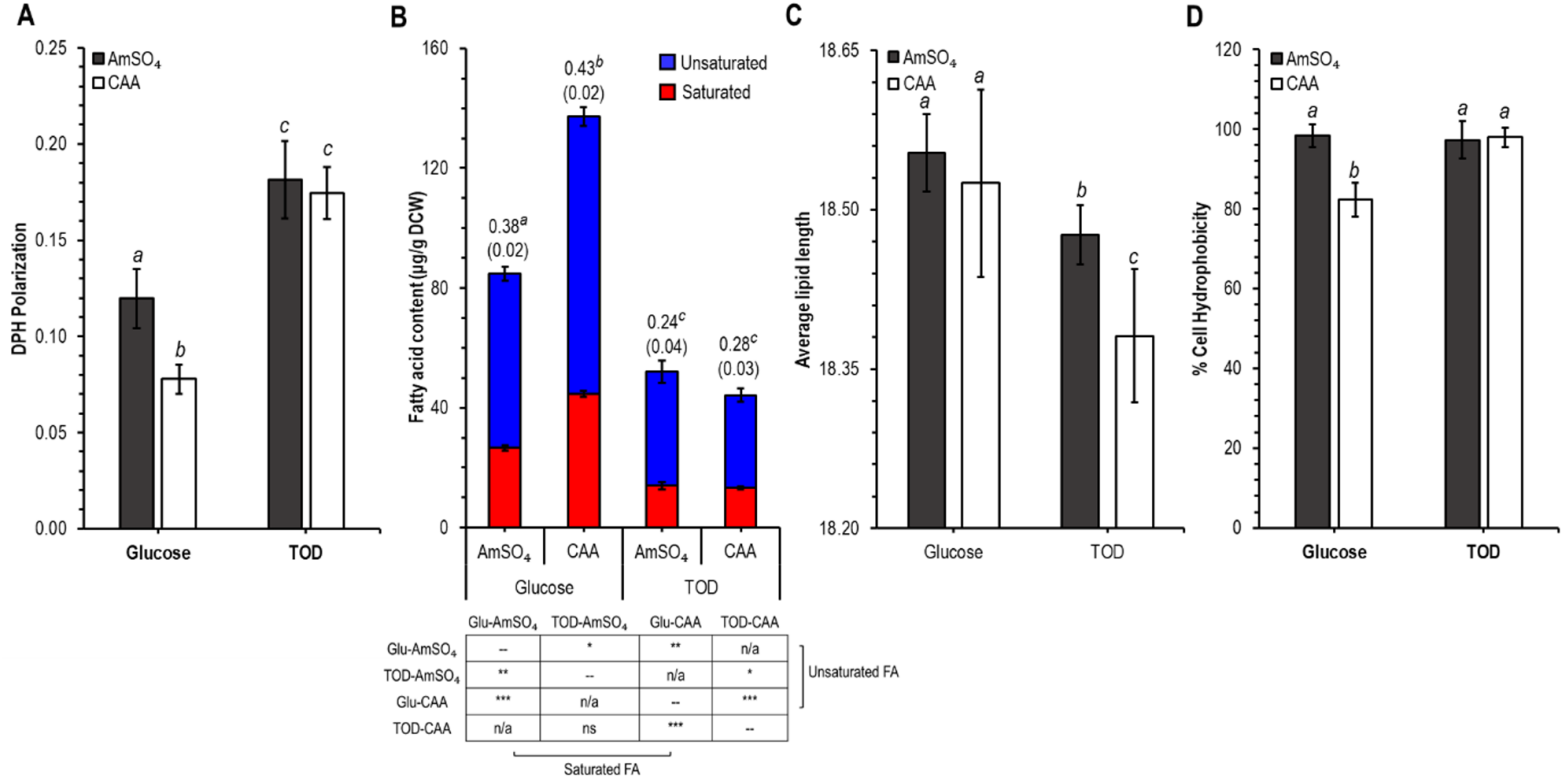
Membrane polarization (**A**), fatty acid content (**B**), average lipid length (**C**), and cell surface hydrophobicity (**D**) of *C. maltosa* cells grown in glucose or TOD with AmSO_4_ or CAA. Values above the bars in (**B**) indicate the saturated:unsaturated fatty acid ratios with their standard deviation in parentheses. Bars marked with different letters indicate a significant difference. Three biological replicates were used in these experiments, and the significant changes relative to the AmSO_4_ condition are indicated as follows: *, *p* < 0.05; **, *p* < 0.001; ***, *p* < 0.0001

The cell membrane is a protective barrier that allows the trafficking of nutrients and wastes in and out of the cell, respectively (Claus et al. 2019). Adjustments to the composition and physiology of the membrane could possibly improve assimilation of the hydrophobic compounds. One such possible change is membrane fluidity, which is dependent upon lipid tail distribution (Belo 2010). We measured DPH polarization as a proxy for membrane fluidity, where fluidity and DPH polarization are inversely correlated. Here, we observed that the fluidity of cells grown in glucose as the carbon source is different for the two nitrogen sources (Fig. 5A). CAA-grown cells exhibit a significantly more fluid membrane than cells grown with AmSO_4_. When the carbon source is TOD, membrane fluidity decreased for cells grown on both of the nitrogen sources relative to those grown on glucose. Thus, while the membrane fluidity was sensitive to nitrogen source during growth on glucose, there were no changes due to nitrogen source in the presence of TOD.

These trends in fluidity were closely aligned with the fatty acid content of the cells (Fig. 5B). Specifically, we observed that in glucose, CAA-grown cells show a significantly higher fatty acid content compared to AmSO_4_, while in TOD, regardless of nitrogen source, the overall fatty acid content was significantly lower. As with fluidity, nitrogen sources affected the fatty acid content, with CAA-grown cells having a lower fatty acid content compared to AmSO_4_.

Unsaturated fatty acids such as oleic acid can affect membrane properties by introducing kinks into the lipid bilayer and increasing membrane fluidity (Lockshon et al. 2007). The ratio of saturated to unsaturated lipid tails also follows trends similar to the fluidity and total fatty acid content. Specifically, on glucose the relative unsaturated fatty acid content is lower when CAA are used relative to AmSO_4_, but on TOD, there is no observed effect due to nitrogen source. Additionally, cells grown on TOD have a higher relative abundance of unsaturated fatty acids than those grown on glucose.

Another metric of membrane composition is the average lipid length. The trends observed for this metric differed from those for fluidity, total lipid content, and relative abundance of saturated and unsaturated lipid tails. Specifically, there was no variation in lipid length due to nitrogen source in the presence of glucose, but there was an observed nitrogen effect during growth on TOD (Fig. 5C). Additionally, average lipid length was significantly lower during growth on TOD relative to glucose for both nitrogen sources.

Another quantifiable membrane property is the cell surface hydrophobicity (Chen et al. 2018). This metric is a complex function of the relative abundance of lipids, proteins and sugars (Liao et al. 2015). In regards to the effect of nitrogen source, trends in *C. maltosa* hydrophobicity were similar to DPH polarization (Fig. 5D). Specifically, when cells were grown on glucose, the two nitrogen sources were associated with different hydrophobicity values, but no difference was observed during growth on TOD. When we analyze the effect of the carbon source, the hydrophobicity of CAA-grown cells again showed a similar trend to DPH polarization: the value increases. However, unlike with DPH polarization, the hydrophobicity of AmSO_4_-grown cells was similar for the two carbon sources. This is interesting because a low hydrophobicity is generally reported alongside less biosurfactant production and therefore less biodegradation (Prabhu and Phale 2003; Men’shov and Shishkina 2006). The observation of increased hydrophobicity of cells grown in CAA and different carbon sources suggests that cells are perhaps better equipped for sensing environmental conditions and adapting their cellular mechanism to the available resources.

Up to this point, the mechanisms and products of the TOD substrate uptake and cell growth remain a black box; although we have previously shown general changes (such as molecular weight distribution) in TOD before and after fermentation with *C. maltosa* (Brown et al. 2023)*,* we have not been able to identify precisely where and for what purpose cells use the TOD substrate, or what the by-products of this utilization are. The complexities of both the carbon source and cellular dynamics add to the complications of this issue. However, the finding that the use of CAA significantly increases fatty acid production in *C. maltosa* is promising for the application and optimization of recycling and upcycling fermentable sugars (Rinki et al. 2024).

## Conclusion

In this study, we demonstrated the importance of nitrogen source selection in the use of *C. maltosa* as a microbial cell factory for the utilization of plastic waste in a possible biorefining platform. We showed that algae extract and casamino acids, representative organic nitrogen sources, supported higher biomass production relative to ammonium sulfate, a representative inorganic nitrogen source, during the utilization of TOD and related model compounds. The magnitude of this increase was disproportional to the associated carbon content of the organic nitrogen, suggesting broader implications beyond simple carbon availability. Possible contributions and mechanisms of the increased biomass production include the mitigation of the energetic demands of assimilation of inorganic nitrogen and the biosynthesis of amino acids and their associated precursors.

This work also provides insight into the possible strategies used by *C. maltosa* to utilize TOD, furthering its position as an intriguing non-model organism. The TOD-specific expression of several low-MW proteins emphasize peroxisomal functionality and the intriguing cell wall canals previously observed during utilization of TOD and alkanes (Erdmann et al. 1989; Zvonarev et al. 2017) as candidates for further research. TOD utilization was also associated with changes in the membrane content and physiology relative to glucose utilization. These changes included a decreased abundance of saturated fatty acid tails relative to unsaturated, consistent with an observed increase in membrane fluidity. The lipid tails were also of a decreased average length.

The system developed in this study could be further strengthened by improving *C. maltosa*’s ability to assimilate TOD. An evolved strain of *C. maltosa* has been successfully isolated using adaptive laboratory evolution and shows a more than 100% increase in specific growth rate (Rodriguez Ocasio et al. In press). The use of nitrogen rich organic substrates paired with an evolved strain can have more impressive growth effects. The opportunity to use microbial cell factories to mitigate, and possibly up-cycle, carbon-rich waste streams such as plastics, provides the opportunity to also utilize nitrogen-rich organic waste streams. We demonstrated that various types and sources of nitrogen can be considered for these bioprocesses, potentially adding value to the waste streams while also diverting these streams from landfills or other problematic means of waste disposal.

## Supplementary Information

Below is the link to the electronic supplementary material.

Supplementary Material 1

## Data Availability

All data generated or analyzed during this study are included in this published article and its supplementary information file.
