# Supplementary material for: Nitrogen management in the optimization of microbial plastic waste upcycling
Source: Bioresour Bioprocess. 2026 Apr 11;13(1):51. doi: 10.1186/s40643-026-01046-z (PMC13070103; doi:10.1186/s40643-026-01046-z)
Supplement: Supplementary file 1 — Supplementary Material 1 [file 40643_2026_1046_MOESM1_ESM.docx]

# NITROGEN MANAGEMENT IN THE OPTIMIZATION OF MICROBIAL PLASTIC WASTE UPCYCLING

Kimia Noroozi^1^, Hong Chen^2^, Vanessa Hupp^1^, Mark A. Blenner^3^, Robert C. Brown^1,4^ Zhiyou Wen^2^, Laura R. Jarboe^1*^

^1^Department of Chemical and Biological Engineering, Iowa State University, Ames, IA, USA, 50011

^2^Department of Food Science and Human Nutrition, Iowa State University, Ames, IA, USA, 50011

^3^Department of Chemical and Biomolecular Engineering, University of Delaware, Newark, DE, 19716, USA

^4^Bioeconomy Institute, Iowa State University, Ames, IA, 50011, USA

# Supplementary Material

Table S1. General composition of amino acids in casamino acids powder (subject to batch-to-batch variability specified by the vendor – US Biological Life Sciences).

| **Amino Acid** | **% wt/wt** | **Amino acid** | **% wt/wt** |
| --- | --- | --- | --- |
| Alanine | 2.8 | Lysine | 7.5 |
| Arginine | 3.6 | Methionine | 2.7 |
| Aspartic acid | 6.3 | Phenylalanine | 4.6 |
| Cysteine | 0.3 | Proline | 9.9 |
| Glutamic acid | 21.1 | Serine | 5.6 |
| Glycine | 2.2 | Threonine | 4.2 |
| Histidine | 2.7 | Tryptophan | 1.1 |
| Isoleucine | 5.6 | Tyrosine | 6.1 |
| Leucine | 8.4 | Valine | 5.0 |

*Table S2. List of model compounds used as the carbon source in this study*

|  |  | Carbon # | Cas number | Molecular weight (g/mol) |
| --- | --- | --- | --- | --- |
| Alcohols | 1-Tetradecanol | 14 | 112-72-1 | 214.39 |
|  | 1-Octadecanol | 18 | 112-92-5 | 270.49 |
|  | 1-Docosanol | 22 | 661-19-8 | 326.61 |
| Alkanes | n-Tetradecane | 14 | 629-59-4 | 198.39 |
|  | n-Octadecane | 18 | 593-45-3 | 254.49 |
|  | n-Docosane | 22 | 629-97-0 | 310.60 |
| Alkenes | 1-Tetradecene | 14 | 1120-36-1 | 196.37 |
|  | 1-Octadecene | 18 | 112-88-9 | 252.49 |
|  | 1-Docosene | 22 | 629-97-0 | 308.58 |
| Esters | Lauryl acetate | 14 | 112-66-3 | 228.38 |
|  | Methyl heptadecanoate | 18 | 1731-92-6 | 284.48 |
|  | Stearyl methacrylate | 22 | 32360-05-7 | 338.60 |
| Carboxylic acids | Myristic acid | 14 | 544-63-8 | 228.38 |
|  | Stearic acid | 18 | 57-11-4 | 284.48 |
|  | Behenic acid | 22 | 112-85-6 | 340.59 |

*Table S3 – Validation of OD_600_ based on DCW measurements for C. maltosa cells grown on TOD. The significant changes relative to the AmSO₄ condition are calculated using an unpaired t test and are indicated as follows: *, p<0.05; **, p<0.001; ***, p<0.0001.*

| Carbon source:  TOD | Nitrogen Source | OD_600_ | DCW (g/L) |
| --- | --- | --- | --- |
| 24 hours | AmSO₄ | 0.48  (0.01) | 0.33  (0.06) |
|  | CAA | 2.63^***^  (0.37) | 1.07^*^  (0.25) |
| 48 hours | AmSO₄ | 1.28  (0.16) | 0.63  (0.25) |
|  | CAA | 1.96^*^  (0.34) | 1.03  (0.15) |


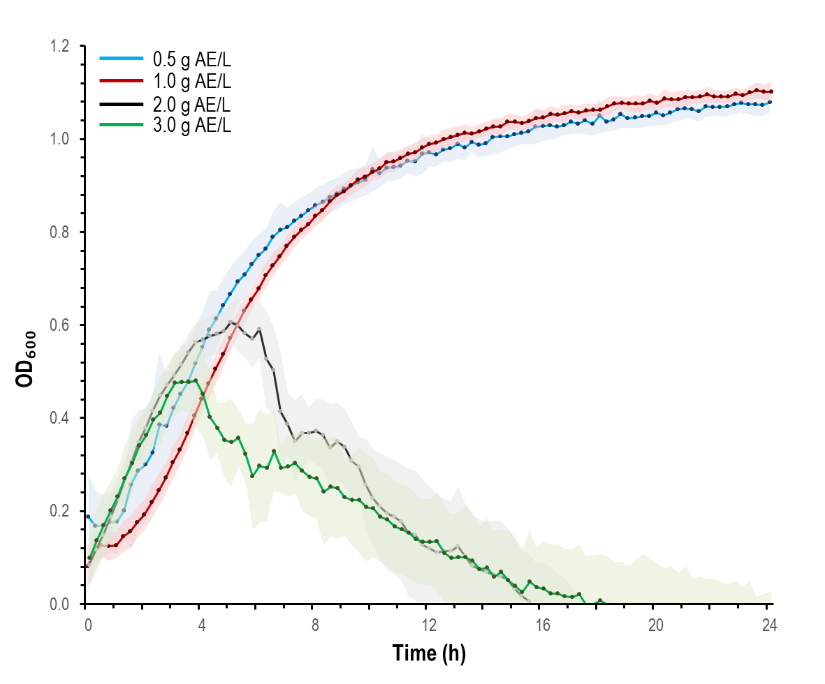


Figure S1. The optimum concentration of algae extract for maximum growth support is 1 g/L.


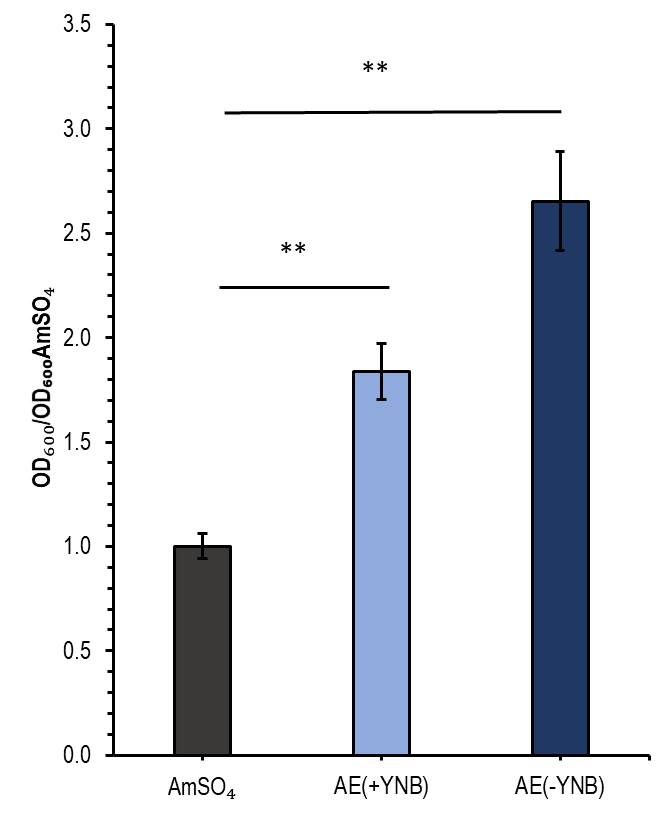


Figure S2. C. maltosa was grown on TOD as the carbon source in media consisting of YNB and ammonium sulfate (AmSO₄), YNB and algae extract ((AE(+YNB)), and just algae extract (AE(-YNB)). While the addition of algae extract as the nitrogen source increases growth compared to AmSO₄, algae extract can be used to substitute all other media requirements (macro- and micro-nutrients) since it is made of whole cell extract. In this case, removing YNB as the source of macronutrients provides a higher increase in growth. The significant changes relative to the AmSO₄ condition are calculated using an unpaired *t* test and are indicated as follows: *, p<0.05; **, p<0.001; ***, p<0.0001.


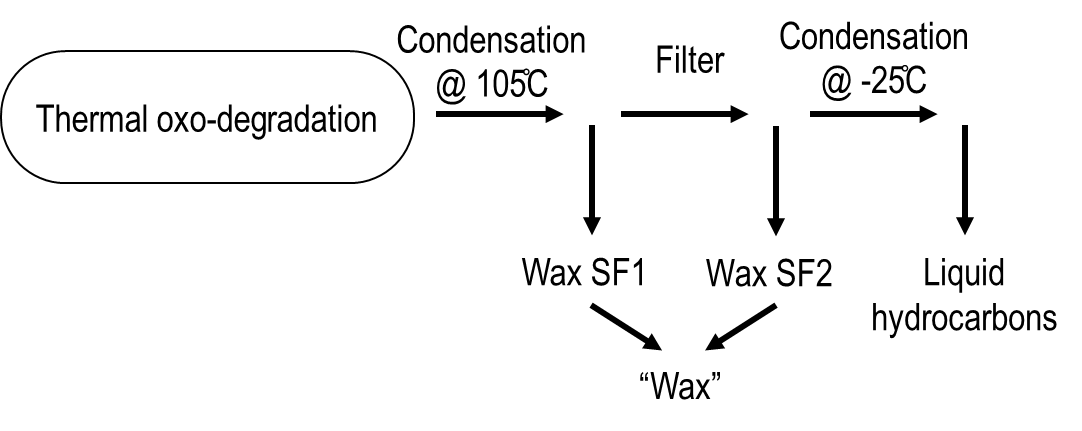


Figure S3. Simplified process schematic adapted from Brown and Rodriguez Ocasio et. al., showing various products and collection conditions of Thermal oxo-degradation


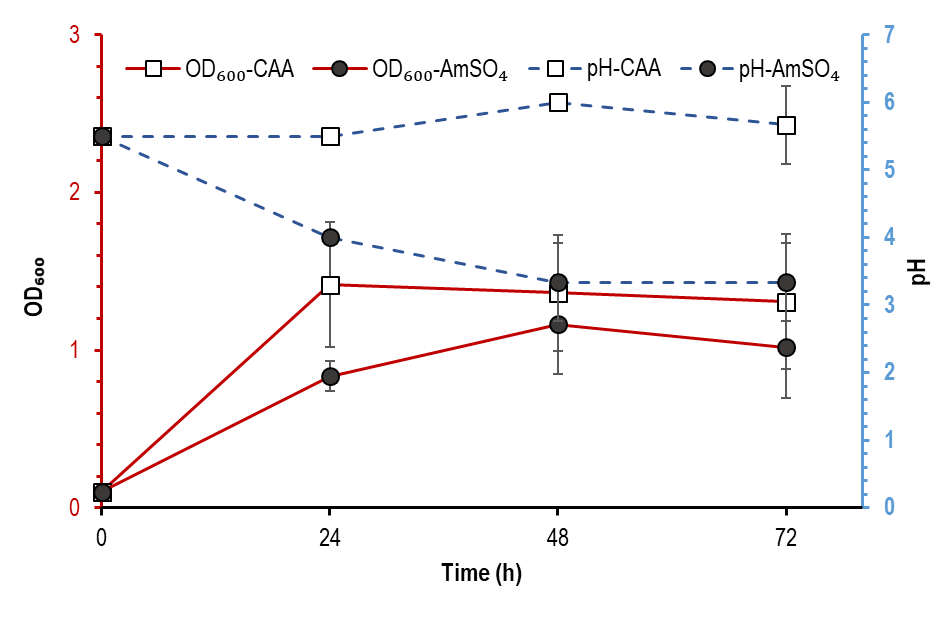


*Figure S4. Media pH during C. maltosa growth in TOD stays relatively stable when casamino acids are used as the nitrogen source. Conversely, the pH follows a decreasing trend in cultures grown on ammonium sulfate. Three biological replicates were used in these experiments, and the significant changes relative to the control condition are indicated as follows: *, p<0.05; **, p<0.001; ***, p<0.0001.*
